# Regulation of Nurses' Hours

**Published:** 1920-11-20

**Authors:** 


### THE GENERAL NURSING COUNCIL MEETING.

### Regulation of Nurses' Hours.

THE monthly meeting of the General Nursing Council was held on November 12, at 2 P.M., at the Ministry of Health. Mr. Priestley, K.C., was in the chair, and the following members were present: Lady Hobhouse, Mrs. Eustace Hills, Miss Tuke, Dr. Bostock Hill, Dr. Bedford Pierce, Dr. Goodall, Sir Jenner Verrall, Miss Lloyd Still, Miss Cox Davies, Miss Seymour Yapp, Mrs. Bedford Fenwick, Miss MacCallum, Miss MacDonald, Miss Peterkin, Miss Smith, Miss Sparshott, Miss Cattell, Miss Dowbiggin, Miss Coulter, Miss Suiss, and Miss Villiers.

The minutes of the last meeting were read, including matters which had been discussed *in camerâ*. The question was raised by Miss Seymour Yapp as to how the report in the *Daily Herald* regarding the voting in the Hours of Employment Bill had been circulated (see THE HOSPITAL, November 6), and Lady Hobhouse and Mr. Priestley expressed themselves very strongly on the matter.

After this business was concluded, Sir Jenner Verrall announced that ninety candidates had applied for the position of Registrar's assistant. The applications and testimonials had been carefully examined, and three candidates were finally interviewed. Eventually Miss Parsloe, trained at Kensington Infirmary, had been selected.

Miss DOWBIGGIN said how glad she was that a candidate trained at a Poor-law infirmary had been chosen, as it was yet another proof that the infirmary nurse was able to hold her own with the hospital nurse.

Miss Parsloe was then summoned, and as neither she nor the Council had any questions to ask each other, the Chairman congratulated her upon her appointment. Miss COX DAVIES then congratulated the Council upon having secured Miss Parsloe, and Miss SEYMOUR YAPP welcomed her in the name of the Poor Law.

Various correspondence was then dealt with by the CHAIRMAN, including letters from the Scottish Board of Health asking that the words "intermediate nurse" might be substituted by "a nurse trained before the issue of the Rules" (the words "intermediate nurse" having a special meaning in Scotland), and that the words "registered nurse" might be substituted by the words "registered general nurse." These matters were referred to the Registration Committee.

Mr. PRIESTLEY then read further correspondence from the Scottish Nursing Council asking whether existing cottage nurses would be placed on the general Register by the English Council, or whether they would consider a supplementary Register for future and existing cottage nurses. The letter pointed out that District Associations are unable to afford the services of a fully-trained nurse, and that the cottage nurses did very useful work.

Mrs. BEDFORD FENWICK protested that it was simply a proposal to perpetuate a poorly trained class of nurse who was also a midwife, and therefore had her status on the Midwives' Roll. The purpose of registration was to raise the status of the trained nurse—not to distinguish between persons who could or could not afford to pay a fully-trained nurse. Every nurse must be efficient, whether for the poor or whether for the rich. To agree to a supplementary Register of partially-trained women was a bad policy, and Mrs. Bedford Fenwick hoped that the Council would absolutely oppose a cottage nurses' Register. Cottage nurses were not qualified for the English Register unless they had served the minimum year in a hospital or Poor-law infirmary.

Miss SEYMOUR YAPP hoped that cottage nurses would not be included in a supplementary Register, "although," she said, "they are very valuable people, because they will do the domestic work which the fully-trained nurse will not do." The cottage nurse was required to be a midwife, and was therefore already provided for.

Miss PETERKIN maintained that the Council should not have a special Register for cottage nurses.

Miss CATTELL said that the Council was a progressive one, and that the entrance to registration was quite wide enough already.

Lady HOBHOUSE said that as the Council had previously decided not to admit cottage nurses on the Register, they could not now go back on it. In any case, however, she would like to place on record that she was very glad that Scotland and Ireland had admitted them. In this country, she thought, the serious difficulties which were bound to arise with regard to nursing in rural districts would have largely to be met by affiliation.

Mr. PRIESTLEY read the proposed reply to the Scottish Board to the effect that the Council did not see their way to form a supplementary part of their Register for cottage nurses.

Miss VILLIERS then proposed that the consideration of the letter and enclosures from the Ministry of Labour be not further discussed *in camerâ*. She added that she had received newspaper comments on the matter having been discussed in private at the previous meeting, and she understood that there was strong feeling on the matter on the part of some of the nursing profession.

She was seconded by Miss COX DAVIES, who maintained that the matter should be discussed openly before the Press rather than that diluted information should be given to them from another source.

Miss SEYMOUR YAPP said she would like to support Miss Villiers. "The Council," she added, "is fortunate in having members who are interested in nursing papers. Surely it is possible for the Press to be asked to abstain from reporting anything we do not want reported, because by remaining they get the sense of the meeting. We are in the hands of the Press, because we rely upon them largely for our nurses' education, and useful articles on registration have appeared in our nursing papers."

Mr. PRIESTLEY said by a rule of Council the meetings were to be made public, unless any member requested that certain matters should be discussed *in camerâ*.

Miss Villiers' resolution was then put to the vote and carried.

Dr. BEDFORD PIERCE moved that the Minister of Health should be asked to introduce a Bill to regulate the hours of nurses employed in hospitals or other institutions for the care of the sick. He said that at the last meeting the Council were in the rather unfortunate position of being asked to vote upon the inclusion of nurses in a Bill which they had not seen. He voted against that inclusion because he did not think it was in the real interest of nurses that they should be included. He was, however, most anxious that the conditions of service of nurses should be greatly improved, and he did not consider that the nurses received the emoluments or the treatment at the hands of the public which the character of their services demanded. There were great difficulties to be overcome, especially as regards the private nurse, and he thought that the Minister of Health was the proper person to make inquiries into these difficulties because he was interested in the health of the community and the nurses' services to that community. Therefore he asked that the Minister should be requested to bring in this Bill.

Miss SPARSHOTT said that she had opposed nurses being included in the Minister of Labour's Bill, but she thought it was essential that their hours should be regulated.

Miss LLOYD STILL said that she was in entire sympathy with the proposal, because she wanted to shorten the nurses' hours and improve their conditions in every way; but in the best interests of the public, of the sick, and of the nurses themselves, she thought it absolutely wrong that they should be included in the Bill under the Ministry of Labour.

Miss MACCALLUM opposed the resolution, as it created a procedure and left the affairs of the nursing profession in

**The General Nursing Council Meeting**—(*continued*).

the hands of one man. The Minister of Health of the future might wish to lengthen the hours of the nurses if there were a shortage of nurses.

Mrs. BEDFORD FENWICK said that the Governors of hospitals were a law unto themselves, and nurses should be included in the Labour Bill, because it would act as a lever on their employers and compel them to reduce their hours.

Miss LLOYD STILL replied that the Governors of hospitals did all they could to protect their nurses.

Dr. BOSTOCK HILL said that he had not been present at the last meeting, but apparently the Council had definitely decided against the inclusion of nurses in the Labour Bill. If that was the case, it seemed to him that the resolution proposed by Dr. Bedford Pierce was a very good one. It did not propose that the Minister of Health should act as an autocrat, but that the Bill should prevent any over-pressure of the nurse.

Dr. GOODALL said he had put the matter to his nurses, and found that they would prefer to come under the Minister of Health than the Minister of Labour. There was no reason to suppose that the Minister of Health would be more autocratic or shorter-lived than the Minister of Labour.

Miss COX DAVIES was also in agreement with the regulation of nurses' hours, and she had found that the hospital committees were only too ready to help their nurses.

The resolution was put to the meeting and carried by 14 votes to 6.

Miss COX DAVIES proposed an amendment to the effect that the number of working hours should be limited to forty-eight.

She was seconded by Miss PETERKIN.

The amendment was carried, Mrs. Bedford Fenwick and other members abstaining from voting. Mrs. BEDFORD FENWICK explained that she approved of forty-eight hours as a maximum, but not of the resolution of Dr. Bedford Pierce.

Other matters concerning the framing of the rules were then dealt with, also the certificate and the seal were handed round for approval, and Mrs. Eustace Hills requested to assist the Registration Committee with advice as regards the lettering on the certificate of registration. The certificate, which allows for the signature of the Chairman, the Chairman of the Registration Committee, and the Registrar, was approved as regards wording. Mrs. BEDFORD FENWICK added that it would be enlarged a little to allow of a margin in order that the nurse might be able to have it framed.

Miss LLOYD STILL, Chairman of the Education and Examination Committee, moved that the interim reports of the Committee be considered and approved. These dealt with the general education of nurses, the preliminary schools, the terms of training, and so forth.

Sir JENNER VERRALL read the report of the Finance Committee, and announced that it was hoped that office accommodation had been secured at York Gate, Regent's Park, at a rent of £325 per annum, on a lease of three years.

The meeting then closed.

